# Serum 4β-hydroxycholesterol increases during fluconazole treatment

**DOI:** 10.1007/s00228-020-03041-5

**Published:** 2020-11-17

**Authors:** Dieter Lütjohann, Frans Stellaard, Anja Kerksiek, Jörn Lötsch, Bruno G Oertel

**Affiliations:** 1 Institute of Clinical Chemistry and Clinical Pharmacology, University Hospital Bonn, Venusberg-Campus 1, 53127 Bonn, Germany; 2 Institute of Clinical Pharmacology, Goethe-University Frankfurt, Theodor Stern Kai 7, 60590 Frankfurt, Germany; 3 Fraunhofer Institute for Molecular Biology and Applied Ecology IME, Branch for Translational Medicine and Pharmacology TMP, Theodor Stern Kai 7, 60590 Frankfurt, Germany

**Keywords:** Oxysterols, Cholesterol metabolism, Antifungal, Cytochrome P450, Bile acid precursors, Cholesterol synthesis

## Abstract

**Purpose:**

The antifungal drugs ketoconazole and itraconazole reduce serum concentrations of 4β-hydroxycholesterol, which is a validated marker for hepatic cytochrome P450 (CYP) 3A4 activity. We tested the effect of another antifungal triazole agent, fluconazole, on serum concentrations of different sterols and oxysterols within the cholesterol metabolism to see if this inhibitory reaction is a general side effect of azole antifungal agents.

**Methods:**

In a prospective, double-blind, placebo-controlled, two-way crossover design, we studied 17 healthy subjects (nine men, eight women) who received 400 mg fluconazole or placebo daily for 8 days. On day 1 before treatment and on day 8 after the last dose, fasting blood samples were collected. Serum cholesterol precursors and oxysterols were measured by gas chromatography-mass spectrometry-selected ion monitoring and expressed as the ratio to cholesterol (R_sterol).

**Results:**

Under fluconazole treatment, serum R_lanosterol and R_24,25-dihydrolanosterol increased significantly without affecting serum cholesterol or metabolic downstream markers of hepatic cholesterol synthesis. Serum R_4β-, R_24S-, and R_27-hydroxycholesterol increased significantly.

**Conclusion:**

Fluconazole inhibits the 14α-demethylation of lanosterol and 24,25-dihydrolanosterol, regulated by CYP51A1, without reduction of total cholesterol synthesis. The increased serum level of R_4β-hydroxycholesterol under fluconazole treatment is in contrast to the reductions observed under ketoconazole and itraconazole treatments. The question, whether this increase is caused by induction of CYP3A4 or by inhibition of the catabolism of 4β-hydroxycholesterol, must be answered by mechanistic in vitro and in vivo studies comparing effects of various azole antifungal agents on hepatic CYP3A4 activity.

## Introduction

**Fig. 1:**
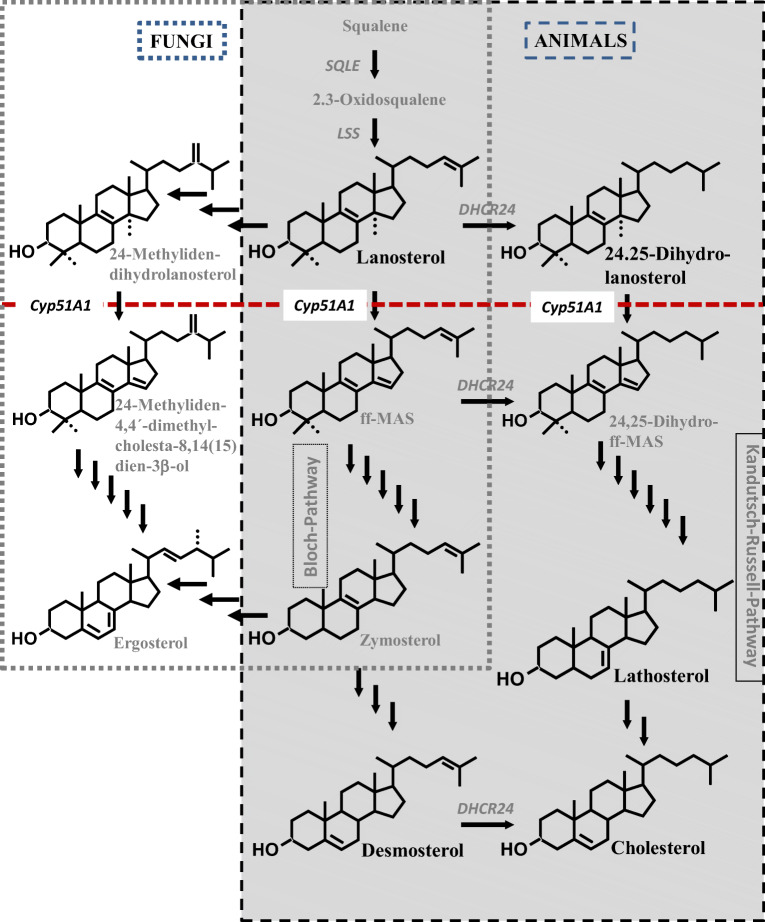
The metabolic pathway converting lanosterol into ergosterol in fungi (....) and into cholesterol in animals (- - -). The red dotted line symbolizes the inhibitory effect of azoles during CYP51A1-regulated 14-demethylation. SQLE, squalene epoxidase; LSS, lanosterol synthase; ff-MAS**,** follicular fluid meiosis-activating sterol or 4,4′-dimethylcholesta-8,14(15),24-trien-3β-ol; DHCR24, 24-dehydrocholesterol reductase

**Fig. 2:**
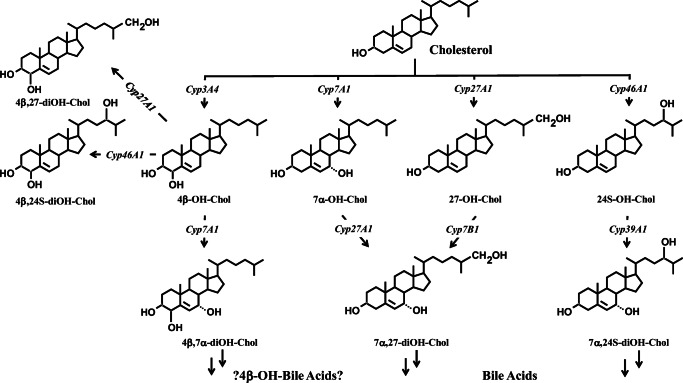
CYP450-regulated metabolism of cholesterol into oxysterols. OH-Chol, hydroxycholesterol; diOH-Chol, dihydroxycholesterol

Systemic antifungal azoles are inhibitors of cytochrome P450 (CYP) isozymes, such as CYP3A4/A5/A7, CYP2C9/C19, and CYP51A1 to varying degrees [1–3]. Potent inhibitors of CYP3A4 can significantly increase the plasma concentrations of the active forms of drugs such as the hydroxymethyl-glutaryl coenzyme A reductase inhibitors simvastatin, lovastatin, and atorvastatin or the hypnotic benzodiazepines midazolam or triazolam, which are mainly metabolized via CYP3A4 [4–6]. The antifungal activity of the azoles is based on the inhibition of the CYP51A1-dependent 14α-demethylation of lanosterol or 24-methyliden-dihydrolanosterol producing ergosterol (Fig. 1) [7–9]. In humans, lanosterol and 24,25-dihydrolanosterol are also 14α-demethylated as an early metabolic step in the steroidal part of cholesterol synthesis (Fig. 1) [10, 11]. The 4β-hydroxylation of cholesterol is dependent on CYP3A4 (Fig. 2) [12]. Serum 4β-hydroxycholesterol has been proposed as an indicator of CYP3A4 activity [13–15]. Rifampicin is an inductor of CYP3A4, diagnosed by increased serum concentrations of 4β-hydroxycholesterol [16, 17]. We previously showed that 4β-hydroxycholesterol was lowered in serum from itraconazole-treated patients [18]. CYP3A4 inhibition and reduction of serum 4β-hydroxycholesterol have also been shown during ketoconazole treatment [19]. It must be questioned whether or not the inhibition of 4β-hydroxylation of cholesterol is a general side effect of antifungal azoles. Therefore, we tested the effect of fluconazole on serum 4β-hydroxycholesterol and other oxysterols.

## Material and methods

### Study enrolment and design

Potential study candidates carrying genetic variants CYP2C9*2, CYP2C9*3, CYP2C19*2, CYP2C19*3, CYP2C19*4, CYP3A5*3, and *OPRM1* 118A>G (rs1799971) were excluded from the study, based on established pyrosequencing assays [20–22]. This was done because these variants may have confounded the effects of fluconazole. The volunteers carried the CYP3A5*1 allele homozygously and can therefore be considered CYP3A5 expressors in all probability, although neither CYP3A4/5 phenotyping nor complete gene sequencing was performed. The subjects’ actual health was determined by medical history, physical examination including vital signs, and routine clinical laboratory test results.

Nine male and eight female healthy subjects were finally enrolled. The data concerning the age, weight, height, and body mass index (BMI) of the males and females are shown in Table 1. Enrolled subjects participated in two study periods, which were separated by a washout period of at least 2 weeks. In each study period of a prospective, double-blind, placebo-controlled, two-way crossover design, subjects received for 7 days oral doses of either 400 mg fluconazole (two capsules of Fluconazol STADA® 200 mg, STADApharm GmbH, Bad Vilbel, Germany) or placebo (two capsules containing Füllstoff DAC® according to the Deutscher Arzneimittel-Codex), once daily at 8 a.m. The actual experiments took place on day 8 of each period starting 2 h after the last dose of fluconazole as described previously [20]. During treatment with fluconazole, the diet of the participants was not monitored. However, it is likely that possible dietary effects were random across placebo and fluconazole conditions, so a bias in the results by a particular diet seems unlikely.

**Table 1:** Subject characteristics

	Age (years)	Weight (kg)	Height (cm)	BMI (kg m^−2^)
All	26 ± 3^a^	72 ± 10	177 ± 8	23 ± 2
Males (*n* = 9)	27 ± 3	78 ± 7	183 ± 5	24 ± 3
Females (*n* = 8)	26 ± 4	64 ± 7	170 ± 6	22 ± 2
Males vs. females	NS	*P* < 0.01	*P* < 0.01	NS

^a^Values represent the mean ± SD. Differences between males and females were tested using the Mann-Whitney *U* test

Venous blood samples were taken in serum monovettes on day 1 prior to administering the first dose and on day 8 2 h after administering the last dose of fluconazole/placebo. The samples were immediately centrifuged at 3000*g*, and the serum supernatant was subsequently frozen and stored at − 80 °C pending further analysis. In a period lasting from 30 days prior to the first study period to the end of the second and last study periods, subjects were prohibited to take any other medication except the study medication. Alcohol intake was prohibited during the actual study periods but not during the washout periods. Food and beverages were prohibited on day 1 and day 8 of each study period but only until the blood sample was taken.

In the same study, the subjects’ sense of smell and its changes after the administration of fluconazole were also tested. The result was negative and was published separately and not redundant to this report [23]. In addition, the consequences of cytochrome P450 reductase inhibition by fluconazole on opioid-induced analgesia in humans were addressed in a translational approach, also with a negative result [20]. The latter is the reason for the *OPRM1* genotyping; however, as the opioid remifentanil had been administered only once at the end of the fluconazole ingestion period and no interactions with cholesterol metabolism are known for this drug, an influence on the present results seems unlikely. Specifically, the remifentanil infusion of the opioid was started 2 h after the last dose of 400 mg fluconazole. The exact timing of the study procedures including administration of the medication is shown in the first figure in Oertel et al. [20]. Of note, the two earlier reports on other parts of the project results mentioned above are not redundant for the present evaluations.

### Analysis of serum concentrations of sterols, oxysterols, bile acids, and fluconazole plasma concentrations

Serum concentrations of cholesterol were quantified using gas chromatography (GC)-flame ionization detection with 5α-cholestane as an internal standard [24, 25]. Quantification of the cholesterol precursors lanosterol, 24,25-dihydrolanosterol, desmosterol, and lathosterol (surrogate markers of the endogenous cholesterol synthesis rate) and the plant sterols campesterol and sitosterol (surrogate markers of cholesterol absorption) was performed by highly specific and sensitive GC-mass spectrometry-selected ion monitoring (GC-MS-SIM) with epicoprostanol as an internal standard [24, 25]. The oxysterols 4β-, 7α-, 24S-, and 27-hydroxycholesterol were quantified by an isotope dilution GC-MS-SIM methodology using the corresponding deuterium-labeled oxysterols as internal standards [25]. The serum bile acids cholic acid, deoxycholic acid, chenodeoxycholic acid, lithocholic acid, and ursodeoxycholic acid were quantified by an isotope dilution GC-MS-SIM method according to [26].

Fluconazole plasma concentrations were analyzed by liquid chromatography-tandem mass spectrometry on an API 4000 triple quadrupole mass spectrometer with a turbo V source (AB Sciex, Darmstadt, Germany) operated in the positive mode as reported earlier [20].

### Statistical analysis

The data and statistical analysis comply with the recommendations on experimental design and analysis in pharmacology [27]. The concentrations of the non-cholesterol sterols and oxysterols were corrected for the cholesterol concentration and expressed as R_parameter. The same subjects were studied under fluconazole treatment and placebo treatment before and after 8 days of treatment. This comparison is the primary parameter of the study. Unless otherwise stated, values reported are means ± SD. The changes under fluconazole treatment were compared with the changes under placebo treatment. Due to the small sample sizes and lacking knowledge about the data distribution, the Wilcoxon-paired test was applied to test the changes under fluconazole and placebo treatments [28]. Additionally, with the Mann-Whitney *U* test, it was tested whether or not the changes were different for males and females [29]. Differences between the baseline concentrations before fluconazole treatment and before placebo treatment were tested with the Wilcoxon-paired test to exclude a bias between the two testing periods. These statistical tests were performed using GraphPad Prism (GraphPad software, version 5.00, San Diego, USA). The α-level was set at 0.05.

Finally, to investigate the observed sex differences in the effects of fluconazole on cholesterol metabolism, plasma concentrations of fluconazole were compared between the sexes. To this end, the three fluconazole concentrations at the three sampling points on day 8 were subjected to analysis of variance for repeated measurements, with “time” as the within-subjects factor and “sex” as the between-subjects factor. This analysis was performed with the statistical software package SPSS (version 26 for Linux, IBM, Chicago, IL, USA).

## Results

**Fig. 3:**
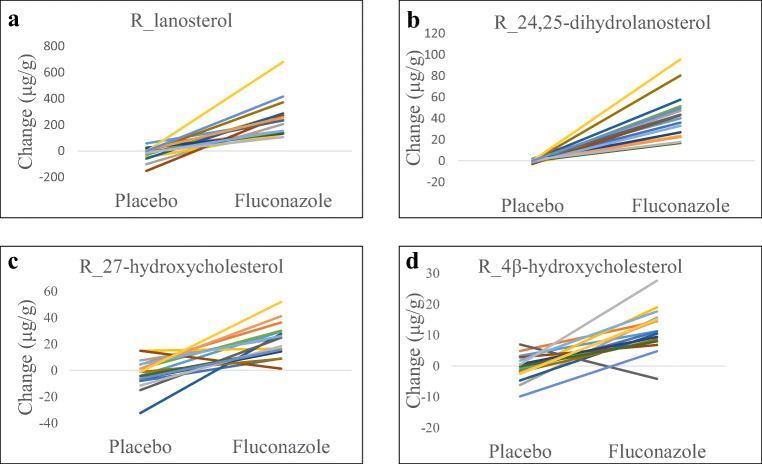
The changes of cholesterol-corrected lanosterol (**a**), 24,25-dihydrolanosterol (**b**), 27-hydroxycholesterol (**c**), and 4β-hydroxycholesterol (**d**) after 8 days of placebo and fluconazole treatments. The lines connect the placebo and fluconazole data of individual subjects

The subject characteristics are comprised in Table 1. Females were significantly smaller and lighter than males. The BMIs were not significantly different. In Table 2 and Fig. 3, the changes in measured concentrations of lanosterol, 24,25-dihydrolanosterol, lathosterol, and desmosterol as intermediate metabolites as well as the oxysterols are expressed corrected for the cholesterol concentration. The changes are calculated as the concentration parameter on day 8 minus that on day 1, for both fluconazole treatment and placebo treatment.

**Table 2:** The changes between the cholesterol-corrected concentrations on day 8 and day 1 for both the fluconazole and placebo treatments

		Fluconazole	Placebo	Wilcoxon *P* value
R_lanosterol (μg g^−1^)	All	253 ± 139^a^	− 20 ± 50	< 0.001
	Males	284 ± 168	− 8 ± 45	< 0.01
	Females	218 ± 97	− 34 ± 54	< 0.01
R_24,25-dihydrolanosterol (μg g^−1^)	All	42 ± 21	− 0.2 ± 1.3	< 0.001
	Males	42 ± 23	0.2 ± 1.3	< 0.01
	Females	41 ± 22	− 0.6 ± 1.2	< 0.01
R_lathosterol (mg g^−1^)	All	− 0.13 ± 0.28	− 0.13 ± 0.64	NS
	Males	− 0.24 ± 0.21	0.06 ± 0.30	< 0.01
	Females	0.00 ± 0.31	− 0.34 ± 0.86	NS
R_desmosterol (mg g^−1^)	All	0.11 ± 0.15	− 0.05 ± 0.16	NS
	Males	0.10 ± 0.13	0.01 ± 0.15	NS
	Females	− 0.12 ± 0.18	− 0.11 ± 0.16	NS
Cholesterol (g L^−1^)	All	− 8.8 ± 8.1	− 0.2 ± 14.1	NS
	Males	− 4.9 ± 8.0	− 3.6 ± 9.6	NS
	Females	− 13.3 ± 6.0	3.6 ± 18	0.05
R_7α-hydroxycholesterol (μg g^−1^)	All	− 0.2 ± 4.9	− 1.9 ± 7.3	NS
	Males	− 0.5 ± 5.5	2.1 ± 3.6	NS
	Females	0.05 ± 4.4	7.1 ± 7.6	< 0.05
R_27-hydroxycholesterol (μg g^−1^)	All	23 ± 13	− 2.65 ± 11.3	< 0.001
	Males	28 ± 14	− 0.8 ± 9.0	< 0.01
	Females	18 ± 10	− 4.5 ± 13.8	< 0.05
R_24S-hydroxycholesterol (μg g^−1^)	All	3.0 ± 3.1	− 0.5 ± 2.4	< 0.01
	Males	2.9 ± 3.5	0.3 ± 1.3	NS
	Females	3.2 ± 2.7	− 1.4 ± 3.1	< 0.01
R_4β-hydroxycholesterol (μg g^−1^)	All	12 ± 7	− 0.7 ± 4.0	< 0.01
	Males	11 ± 7	− 1.1 ± 5.3	< 0.05
	Females	12 ± 7	− 0.3 ± 2.3	< 0.01

^a^Values represent the mean ± SD. Statistical differences are calculated using the Wilcoxon-paired test

**Fig. 4:**
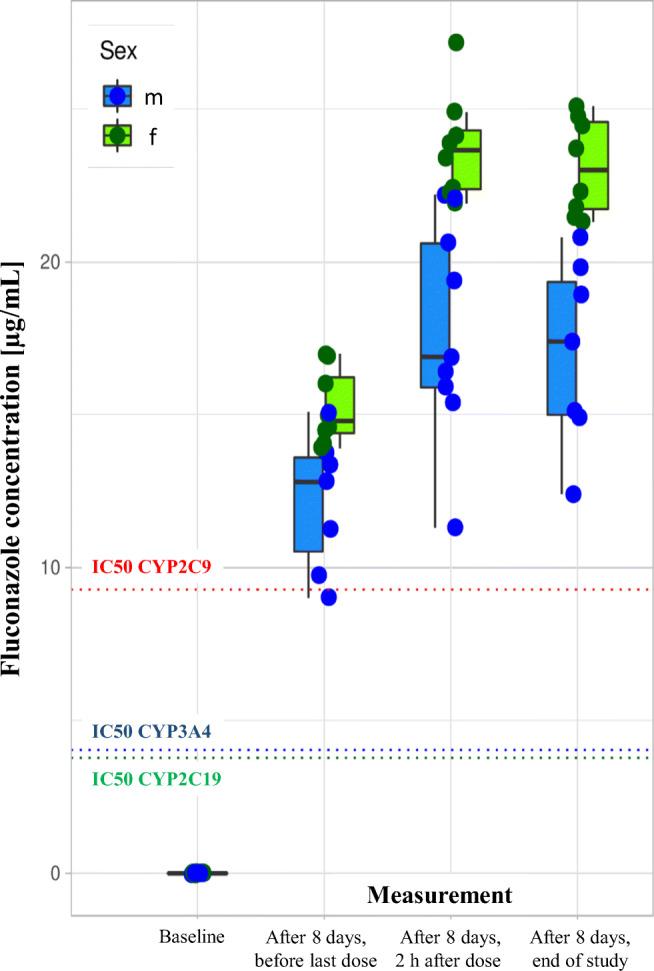
Fluconazole plasma concentrations, separately for the three time points of assessment and separately for men and women. The dotted lines indicate the IC_50_ values for CYP inhibition according to Niwa et al. [38]

The absolute serum concentrations of cholesterol and non-cholesterol sterols and oxysterols and bile acids measured on day 1 before fluconazole treatment were not statistically different from the concentrations on day 1 before placebo treatment. For the cholesterol concentrations, the changes under fluconazole treatment vs. placebo treatment were significant for females, but not for males. This can be ascribed to a larger reduction in concentration in females (*P* = 0.02740). Also, for R_7α-hydroxycholesterol, R_24S-hydroxycholesterol, and R_lathosterol, one of both sexes did not respond significantly differently under fluconazole treatment when compared to placebo treatment (Table 2). The sex-dependent response for R_7α-hydroxycholesterol is caused by a significantly larger increase in females compared to males during placebo treatment. Under fluconazole treatment, males and females did not differ in response. For R_24S-hydroxycholesterol and R_lathosterol, males and females did not differ in response to both the fluconazole and placebo treatments. For all other parameters, no sex differences were seen, not under fluconazole nor placebo treatment. For R_lathosterol, the difference between the fluconazole and placebo treatments was only significant for males. For the whole group, significant fluconazole effects were observed for R_lanosterol, R_24,25-dihydrolanosterol, R_27-hydroxycholesterol, R_24S-hydroxycholesterol, and R_4β-hydroxycholesterol. In all cases, the cholesterol-corrected concentrations increased. The fluconazole effects were also studied for the cholesterol absorption markers R_campesterol and R_sitosterol and the serum concentrations of individual total bile acids (cholic acid, chenodeoxycholic acid, and deoxycholic acid) and its sum (data not shown). No fluconazole effects were found. The percentage increase for the parameters that changed significantly is comprised in Table 3. The plasma concentrations of fluconazole were significantly higher in women than in men (Fig. 4).

**Table 3:** The mean percentage increase in concentration under fluconazole treatment corrected for the placebo effect

	Increase (%)
R_lanosterol	356
R_24,25-dihydrolanosterol	3477
R_27-hydroxycholesterol	25
R_24S-hydroxycholesterol	9
R_4β-hydroxycholesterol	48

## Discussion

Our data show similar increases of serum lanosterol and 24,25-dihydrolanosterol due to inhibition of the 14α-demethylation as seen in our previous study applying itraconazole [18]. In both studies, no significant decreases in lathosterol and desmosterol concentrations were observed. In view of the understanding of cholesterol metabolism, this phenomenon is of interest. The 24,25-dihydrolanosterol is formed from lanosterol (Fig. 2). In the untreated situation, the absolute mean serum concentrations of lanosterol and 24,25-dihydrolanosterol were 17 and 0.3 mg L^−1^, respectively, whereas lathosterol and desmosterol concentrations are around 204 and 156 mg L^−1^, respectively. Under fluconazole treatment, lanosterol and 24,25-dihydrolanosterol concentrations increased to 58 and 7 mg L^−1^, whereas lathosterol and desmosterol concentrations were unchanged, 174 and 145 mg L^−1^. Apparently, lanosterol formation is not a rate determinant for lathosterol and desmosterol formation. In our previous study, itraconazole reduced 4β-hydroxylation of cholesterol [18]. This confirmed the expected inhibition capacity of itraconazole, and it confirmed the validity of 4β-hydroxycholesterol as an indicator of hepatic CYP3A4 activity. In contrast, we surprisingly found that fluconazole did increase absolute serum concentrations of 4β-hydroxycholesterol as well as R_4β-hydroxycholesterol. This result questions the expected inhibitory action of fluconazole and/or the validity of 4β-hydroxycholesterol as a CYP3A4 activity marker. The increased 4β-hydroxycholesterol concentration can be explained by increased production or decreased catabolism of 4β-hydroxycholesterol. Decreased metabolism may be questioned, since the half-life of deuterium-labeled 4β-hydroxycholesterol in healthy subjects is already near 60 h [30]. Unfortunately, we were not able to compare itraconazole and fluconazole treatments in one study in the same subjects. Comparing the two studies, the following differences are observed. Itraconazole was tested in an open, prospective exploratory trial including eight male patients with onychomycosis (mean age 48 years, mean BMI 25 kg m^−2^). It was tested in two 8-day cycles of treatment with 400 mg itraconazole once daily, separated by a washout phase of 20 days [18]. In the present study, 17 healthy subjects (9 males, 8 females) were tested (mean age 26 years, mean BMI 23 kg m^−2^). The same subjects were tested under fluconazole (400 mg day^−1^) and placebo in a prospective, double-blind, placebo-controlled, two-way crossover design with a 2-week washout period [20]. It appears unlikely that onychomycosis affects cholesterol synthesis and metabolism. It is also unlikely that the effect of antifungal treatment on cholesterol metabolism is different in 26- and 48-year-old subjects.

The first principle effect of azoles is the inhibition of 14α-demethylation of lanosterol and 24,25-dihydrolanosterol. The serum R_lanosterol and R_24,25-dihydrolanosterol concentrations increased 14.2- and 400-fold during the first treatment cycle and 8.8- and 150-fold during the second cycle with 400 mg itraconazole given daily within each cycle [18]. Treatment with 400 mg fluconazole daily for 8 days resulted in a 3.6- and 35-fold increase in serum R_lanosterol and R_24,25-dihydrolanosterol, respectively, much lower than those observed for the same dose of itraconazole [20]. Both treatments resulted in unchanged serum levels of R_lathosterol and R_desmosterol. This indicates that hepatic cholesterol synthesis is unaffected by itraconazole and fluconazole treatments. Also, R_campesterol and R_sitosterol as serum markers for cholesterol absorption were unchanged under fluconazole treatment. These findings for cholesterol synthesis and absorption are consistent with the finding that serum total cholesterol was statistically unchanged. However, the *P* value of 0.07 indicated a tendency towards a 5% reduction. Serum R_7α-hydroxycholesterol did not change under both treatments indicating unchanged bile acid synthesis. This observation is consistent with the unchanged serum bile acid concentrations. Serum R_24S-hydroxycholesterol increased by 30% under itraconazole and 9% under fluconazole treatment. The 24S-hydroxycholesterol is exclusively formed in the brain and transported over the blood-brain barrier into the periphery. It is unclear whether increased R_24S-hydroxycholesterol reflects increased formation or inhibited metabolism, i.e., degradation to bile acids in the liver. R_27-hydroxycholesterol increased by 13% under itraconazole and 25% under fluconazole. Enhanced 27-hydroxylation is expected to result in enhanced chenodeoxycholic acid synthesis. Inhibited catabolism of 27-hydroxycholesterol should lead to reduced chenodeoxycholic acid synthesis. Both possible effects were not confirmed by the serum bile acid profile. R_4β-hydroxycholesterol decreased by 20% under itraconazole and increased by 48% under fluconazole treatment. It appears unlikely that this difference between treatment results is due to the differences in experimental protocols. Under fluconazole treatment, males and females showed similar increases in serum R_4β-hydroxycholesterol. Under placebo treatment, the changes in R_4β-hydroxycholesterol were negligible and not different between males and females. Fluconazole treatment increased both serum R_27-hydroxycholesterol and R_4β-hydroxycholesterol. The 27-hydroxylation and 4β-hydroxylation of cholesterol are hepatic processes controlled by different CYP families, being CYP27A1 and CYP3A4, respectively. The mechanisms leading to increased serum levels are unknown. Potentially, the formation of the metabolites can be enhanced or the metabolism of the metabolites inhibited. This aspect should be addressed in in vitro and/or animal studies actually measuring CYP3A4 activity under exposure to different triazole antifungal agents. Also, for the alternative marker for CYP3A4, oral midazolam pharmacokinetics must be included for comparison [31–33]. The benzodiazepines midazolam and triazolam are metabolized during its first pass and elimination processes by CYP3A4 and are thus excellent valid exogenous CYP3A4 probes. The major effects of itraconazole and fluconazole on the pharmacokinetics and pharmacodynamics of intravenous (i.v.) and oral application of the benzodiazepines triazolam and midazolam were carefully investigated by Olkkola and colleagues [5]. After i.v. midazolam, itraconazole (200 mg/day) reduced the mean plasma clearance of midazolam by 70% and fluconazole by 50%. Both itraconazole and fluconazole increased the individual *C*_max_ values by two to six times. The changes in the pharmacokinetics of oral midazolam resulted from an increase in oral bioavailability and a decrease in plasma clearance of midazolam. Thus, the use of large doses of i.v. midazolam increases the risk of clinically significant interactions with antifungal drugs such as azoles. Use of oral midazolam with itraconazole and fluconazole should be avoided [5]. In another study, healthy volunteers received 50 mg, 100 mg, or 200 mg of fluconazole or placebo orally once a day for 4 days. On day 4, they took a 0.25-mg oral dose of triazolam. The pharmacodynamic effects of triazolam were increased significantly (*P* < 0.05) by fluconazole 100 mg and 200 mg. The authors conclude that, when triazolam is used concomitantly with fluconazole 50–200 mg, the dose of triazolam should be reduced accordingly. Simultaneous use of triazolam with higher fluconazole doses should be avoided in order to avoid the marked increase of the hypnotic effect of triazolam [6].

Interestingly, a comparison of 4β-hydroxycholesterol and oral midazolam pharmacokinetics has shown that 4β-hydroxycholesterol is much more sensitive in detecting induction of CYP3A4 than detecting inhibition [19]. In the present study, the detection of increased serum 4β-hydroxycholesterol was not a problem. From the seventeen subjects, sixteen exerted an increased value compared to the placebo value. The question remains, whether fluconazole does not affect the metabolism of other drugs taken by the patient. When the CYP3A4-dependent 4β-hydroxylation is inhibited, but less inhibited than the following metabolic steps, the result may still be expressed as an increased serum R_4β-hydroxycholesterol. In humans, the effects of fluconazole, itraconazole, and potentially also ketoconazole on the serum sterol and oxysterol levels must be determined in the same subjects under the same experimental conditions in order to confirm the results so far observed in separate studies.

**Fig. 5:**
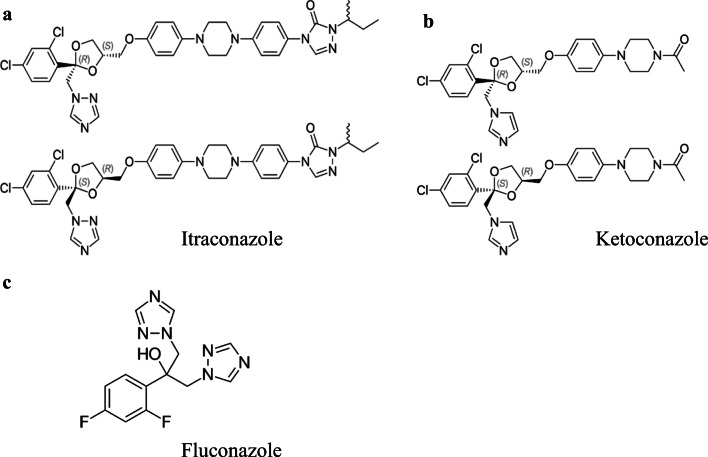
Chemical structures of itraconazole (**a**), ketoconazole (**b**), and fluconazole (**c**)

Itraconazole and fluconazole are triazoles, and ketoconazole is a diazole (Fig. 5). The molecular weights are 706 g mol^−1^ (itraconazole), 306 g mol^−1^ (fluconazole), and 531 g mol^−1^ (ketoconazole). Thus, on a molar basis, the same weight dose represents twice as much fluconazole than itraconazole. The metabolism of the three compounds is different (https://go.drugbank.com/drugs, accessed October 5, 2020). Fluconazole is very poorly metabolized, and over 80% is excreted by the kidneys. Itraconazole and ketoconazole appear as racemic mixtures, and the various isomers are metabolized. Itraconazole is highly converted into some metabolites of which hydroxyitraconazole is the most dominant one. Three to 18% of the parent drug is excreted via the feces, only 0.03% via the urine. Forty percent is excreted in urine as inactive metabolites. Ketoconazole is converted into several inactive metabolites. Approximately 13% of the dose is excreted in the urine, of which 2 to 4% is unchanged drug. The major route of excretion is through the bile into the intestinal tract with about 57% being excreted in the feces. Metabolism of itraconazole and ketoconazole takes place in the liver by CYP3A4, the same enzyme system that they inhibit. They partly regulate their own metabolism. The IC_50_ values for three azoles for inhibition of CYP3A4 are highest for fluconazole (> 200 μM), compared to ketoconazole (1.2 μM) [34] and itraconazole (29 nM) [35]. The binding properties are also different as has been shown by Godamudunage et al. [34] comparing various triazoles including fluconazole and ketoconazole. The authors conclude that CYP3A4 binds some triazoles in more than one orientation, and the orientation can be dependent on the ligand concentration. Putting data together, different effects of different triazoles on 4β-hydroxycholesterol metabolism should not be surprising.

Sex differences were observed for cholesterol, R_lathosterol, R_7α-hydroxycholesterol, and R_24S-hydroxycholesterol. It showed that, under fluconazole treatment, one gender exerted a significantly different response when compared to placebo treatment, whereas the other gender did not. This may mean that both sexes responded differently under fluconazole treatment or under placebo treatment. Data analysis revealed that only the total cholesterol concentration was significantly strongly reduced in females compared to males under fluconazole treatment. Larger population studies are needed to document sex differences.

The present results are in contrast to current information on the role of fluconazole in clinical drug interactions. According to the US Food and Drug Administration (https://www.fda.gov/drugs/drug-interactions-labeling/drug-development-and-drug-interactions-table-substrates-inhibitors-and-inducers, accessed July 7, 2020), fluconazole is a moderate inhibitor of CYP2C9 and CYP3A4 and a strong inhibitor of CYP2C19. Similarly, according to the DrugBank [36, 37] database (https://www.drugbank.com/drugs, accessed July 7, 2020), the target of fluconazole is the yeast CYP51, and interactions with human CYP3A4, 3A5, 2C9, and 2C19 are listed. The only evidence of weak interaction with CYP3A4 comes from an in vitro evaluation with recombinant human CYP3A4 and human fetal CYP3A7, where fluconazole inhibition of CYP3A4 was reported with a value of IC_50_ > 200 μM [34]. However, others reported values of IC_50_ = 13.1 μM for CYP3A4, IC_50_ = 30.3 for CYP2C9, and IC_50_ = 12.3 for CYP2C19 [38]. The closely related values for CYP3A4 and CYP2C9 do not fully match the abovementioned NIH information where fluconazole is listed as a strong CYP2C19 inhibitor but only as a moderate CYP3A4 inhibitor, but they in turn make fluconazole a CYP3A4 inhibitor. As previously reported, the present plasma concentrations of fluconazole were above the IC_50_ values mentioned above at the time of the collection of the present samples for cholesterol metabolite analysis (Fig. 5) [20]. Thus, the lack of CYP3A4 modulation is unlikely due to low plasma concentrations, and since the experiments were conducted under steady-state conditions after 7 days of oral administration of fluconazole, it is also unlikely that locally insufficient concentrations are behind the present observations. Only under the assumption of the high IC_50_ of 200 mM (61.85 mg/mL) the lack of CYP3A4 inhibition could be a problem of drug concentration, but this is probably not of clinical relevance, since 400 mg per day is already a relatively high dose. However, the higher fluconazole plasma concentrations in women than in men provide a straightforward explanation for the more pronounced effects in women (Fig. 4).

Other interactions of fluconazole with drug disposition may be possible in the present context. This concerns transmembrane transporters, which have occasionally been mentioned as interactions with fluconazole. While fluconazole was explicitly denied as an inhibitor of transporters (see the “Introduction” section in [39]), P-glycoprotein (P-gp) is listed in the DrugBank as inhibited by fluconazole. However, the role of these interactions in the present observations on cholesterol metabolism would contrast with the report that P-gp knockout mice maintain cholesterol homeostasis, although these species differences cannot be excluded [40]. A more likely interaction could involve the organic anion transporting polypeptide 1B1 (OATP1B1), at which fluconazole inhibited substance uptake [41]. In fact, OATP1B1 has been observed in mice and human material to play a role in cholesterol homeostasis [42], which therefore may provide an additional explanation for the present results pending specific experiments that include a fluconazole condition in the investigation of the involvement of the transporter in human cholesterol disposition.

## Conclusions

A 1-week short-time treatment with fluconazole resulted in elevated serum levels of R_4β-hydroxycholesterol in healthy subjects. This effect is in contrast to that known for treatment with itraconazole and ketoconazole. After this effect has been demonstrated in a mainly pharmacokinetic analysis in human volunteers, its mechanistic basis still needs to be clarified.
